# The E3 ubiquitin ligase MARCH1 regulates glucose-tolerance and lipid storage in a sex-specific manner

**DOI:** 10.1371/journal.pone.0204898

**Published:** 2018-10-24

**Authors:** Candida Bhagwandin, Erin L. Ashbeck, Michael Whalen, Joanna Bandola-Simon, Paul A. Roche, Adam Szajman, Sarah Mai Truong, Betsy C. Wertheim, Yann C. Klimentidis, Satoshi Ishido, Benjamin J. Renquist, Lonnie Lybarger

**Affiliations:** 1 Cellular and Molecular Medicine, University of Arizona, Tucson, Arizona, United States of America; 2 University of Arizona Cancer Center, University of Arizona, Tucson, Arizona, United States of America; 3 Experimental Immunology Branch, National Cancer Institute, National Institutes of Health, Bethesda, MD, United States of America; 4 Molecular and Cellular Biology, University of Arizona, Tucson, Arizona, United States of America; 5 Mel and Enid Zuckerman College of Public Health, Department of Epidemiology and Biostatistics, University of Arizona, Tucson, Arizona, United States of America; 6 Department of Microbiology, Hyogo College of Medicine, Nishinomiya, Japan; 7 Animal and Comparative Biomedical Sciences, University of Arizona, Tucson, Arizona, United States of America; Tohoku University, JAPAN

## Abstract

Type 2 diabetes is typified by insulin-resistance in adipose tissue, skeletal muscle, and liver, leading to chronic hyperglycemia. Additionally, obesity and type 2 diabetes are characterized by chronic low-grade inflammation. Membrane-associated RING-CH-1 (MARCH1) is an E3 ubiquitin ligase best known for suppression of antigen presentation by dendritic and B cells. MARCH1 was recently found to negatively regulate the cell surface levels of the insulin receptor *via* ubiquitination. This, in turn, impaired insulin sensitivity in mouse models. Here, we report that MARCH1-deficient (knockout; KO) female mice exhibit excessive weight gain and excessive visceral adiposity when reared on standard chow diet, without increased inflammatory cell infiltration of adipose tissue. By contrast, male MARCH1 KO mice had similar weight gain and visceral adiposity to wildtype (WT) male mice. MARCH1 KO mice of both sexes were more glucose tolerant than WT mice. The levels of insulin receptor were generally higher in insulin-responsive tissues (especially the liver) from female MARCH1 KO mice compared to males, with the potential to account in part for the differences between male and female MARCH1 KO mice. We also explored a potential role for *MARCH1* in human type 2 diabetes risk through genetic association testing in publicly-available datasets, and found evidence suggestive of association. Collectively, our data indicate an additional link between immune function and diabetes, specifically implicating MARCH1 as a regulator of lipid metabolism and glucose tolerance, whose function is modified by sex-specific factors.

## Introduction

Metabolic syndrome represents one of the most pressing public health concerns worldwide. A general feature of metabolic syndrome is insulin resistance, hyperinsulinemia, and hyperglycemia [1, 2]. Insulin normally enhances glucose uptake by white adipose tissue and muscle [3], while suppressing hepatic gluconeogenesis [4] and encouraging whole body glucose disposal [5, 6]. Insulin-resistance, in turn, leads to a dysregulation of glucose metabolism and chronically results in overt type 2 diabetes mellitus [1].

Obesity increases the incidence of insulin resistance and type 2 diabetes [7]. Systemic and local (visceral adipose) inflammation has emerged as a key feature of obesity and the progression of type 2 diabetes [8, 9]. Adipocytes produce immune-active molecules, collectively termed adipokines, and adipose tissue contains a remarkable array of resident and infiltrating immune cells that can impact metabolism [10, 11]. Cellular stresses, such as ectopic lipid accumulation and elevated glucose exposure, instigate metabolic pathogenesis [12]. Notably, inflammation exacerbates both hyperglycemia and ectopic lipid accumulation [8, 13, 14]. Obesity increases macrophage and adaptive immune cell (B cell, CD8 T cell, and CD4 TH1 cell) infiltration into visceral adipose tissue, enhancing pro-inflammatory function and a pro-inflammatory cytokine expression profile [15–19]. This inflammatory state depresses insulin sensitivity, whereas anti-inflammatory T regulatory cells (T_regs_) support insulin sensitivity [20].

The E3 ubiquitin ligase MARCH1 (Membrane-Associated RING-CH1; [21]) intersects with inflammation and insulin responsiveness. Its expression is strongest in lymphoid tissues and it functions in antigen-presenting cells (APCs) to negatively regulate, *via* ubiquitin-dependent effects on trafficking, the cell surface levels of Major Histocompatibility Complex class II (MHC-II) molecules and CD86 (a co-stimulatory protein) [21–23]. MARCH1 expression is decreased by APC activation/maturation signals [24, 25] and increased by anti-inflammatory stimuli, such as interleukin-10 [26]. Nagarajan, *et al*. recently showed that MARCH1 can target the insulin receptor for ubiquitin-dependent downregulation, negatively regulating insulin receptor signaling [27]. Accordingly, *in vivo* loss of MARCH1 in liver and adipose tissue correlated with improved glucose clearance, and MARCH1 over-expression impaired clearance [27].

Consistent with this new role for MARCH1, genome-wide association studies (GWAS) have provided modest evidence for an association between MARCH1 and type 2 diabetes. In a GWAS for type 2 diabetes in different Asian populations, *MARCH1* was associated with type 2 diabetes in one ethnic group, but not two others [28]. Another GWAS in a Korean cohort detected an association between *MARCH1* and body-mass index (BMI) [29].

During our experiments to characterize immunity in MARCH1 KO mice, we noted changes in adiposity that prompted us to investigate metabolic parameters. Herein, we report studies conducted to further assess the metabolic phenotype in male and female MARCH1 KO and wildtype mice. We also tested for a genetic association of variants in *MARCH1* with type 2 diabetes in human case-control studies.

## Materials and methods

### Mice

#### Assessment of body weight, adiposity, glucose tolerance, and immune cell infiltration of adipose tissue

Sibling WT and MARCH1 KO experimental mice on the C57BL/6 background were derived through het-het breeding. Mice were reared ad libitum on NIH-31 chow (Harlan Laboratories; 7013). Body and food weights were taken weekly to assess body weight gain and food consumption. All mice were housed in the animal facility at the University of Arizona, and experiments were conducted under the approval and supervision of the Institutional Animal Care and Use Committee (IACUC). Mouse experiments were performed in a blinded fashion with respect to genotype.

Every effort was made to minimize animal suffering, in accordance with the University of Arizona IACUC guidelines. Mice were monitored regularly for any signs of disease and, if noted, veterinary staff were altered and mice promptly euthanized if they displayed signs of disease. Euthanasia was performed in accordance with the methods approved by the American Veterinary Medical Association panel on euthanasia, in which mice were exposed to CO_2_ from a compressed gas cylinder in a closed chamber in the University of Arizona animal facility. All participating personnel received training in the proper use of the CO_2_ chamber. Topical administration of ice-cold ethanol was used for anesthesia prior to blood collection from the tail vein, per IACUC guidelines.

#### Assessment of insulin receptor levels in liver, muscle, and adipose tissue

Littermate male and female C57BL/6 mice or littermate male and female MARCH1 KO mice (approximately 18 weeks old) were used in all tissue isolation experiments. Mice were bred and maintained in-house at the NCI-Frederick animal facility. All mice were cared for in accordance with National Institutes of Health guidelines with the approval of the National Cancer Institute Animal Care and Use Committee. Mice were euthanized by exposure to CO_2_ from a compressed gas cylinder in a closed chamber. All participating personnel received training in the proper use of the CO_2_ chamber.

### Glucose measurements and glucose tolerance tests

Fasting blood glucose and intraperitoneal glucose tolerance tests (IPGTT; 1.5 mg/g body weight) were performed following an 8-hour fast, that was initiated at lights-on. Glucose was measured in blood collected from the tail vein using the FreeStyle Lite glucose monitoring system.

### Collection of stromal-vascular fraction (SVF) of adipose and flow cytometry

Visceral adipose tissue (VAT) was dissociated with collagenase I in KRHB buffer for ~30–45 minutes *a*t 37°C as described [30]. Digested tissue was filtered through a 100 μm cell strainer. Filtrate was centrifuged at 500 x *g* for 5 min, the pellet resuspended in KRHB, and the process repeated 4 times. The final pellet was collected as the SVF. Cell labeling for flow cytometry was performed as described [31]. Cells were analyzed on a FACSCalibur cytometer or an LSRII cytometer (BD Biosciences), and data were analyzed using FlowJo (Treestar). The gating schemes are given in S1 Fig and represent the gates used for quantitation of population percentages and cell numbers (obtained using CountBright Absolute Counting Beads according the manufacturer’s instructions; Life Technologies). The following antibodies were used: CD45.2 (clone 104), CD3 (17A2), CD11b (clone M1/70), MHC-II (clone M5/114.15.2), F4/80 (clone BM8), CD4 (clone L3T4), and CD8 (clone 53–6.7), all from eBioscience, and anti-mouse CD25 (clone PC61) from BioLegend.

### Tissue isolation and immunoblotting

Mouse adipose tissue, skeletal muscle, and liver were isolated and immediately homogenized using a Precellys homogenizer (Bertin Corp.). Cells were solubilized in Triton X-100-containing lysis buffer (10 mM Tris-HCl,pH 7.4, 150 mM NaCl, 1% Triton X-100, 50 mM phenylmethylsulfonyl fluoride, 0.1 mM Nα-tosyl-L-lysine chloromethyl ketone hydrochloride, and 25 mM N-ethylmaleimide) for 1 h at 4°C. The protein content of each sample was determined using a Pierce BCA Protein Assay kit (Thermo Fisher Scientific). Proteins were separated by SDS-PAGE and transferred to PVDF membranes (Bio-Rad) as described previously [32]. Non-specific antibody binding sites on immunoblots were blocked by incubation overnight using 5% nonfat dry milk in PBS containing 0.1% Tween-20 (blotting buffer), washed, and incubated overnight with primary antibodies against insulin receptor β-subunit (Cell Signaling Technology) and appropriate secondary antibodies in blotting buffer. Immunoblots were re-probed using HRP-conjugated anti-GAPDH monoclonal antibody (Cell Signaling Technology) as a loading control. Immunoblots were quantitated and analyzed using a GS-900 calibrated densitometer and the software Image Lab (Bio-Rad) following the manufacturer's instructions. Quantitation of blot intensities was carried out by analysis of data obtained in the linear range of exposure. In each sample the ratio of insulin receptor β-subunit band intensity was expressed relative to the amount of GAPDH present.

### MARCH1 single-nucleotide polymorphism (SNP) analyses

We performed a candidate gene investigation of *MARCH1* SNPs in the following type 2 diabetes case-control datasets: the Northwestern NUgene (Vanderbilt University Medical Center, n = 3,357; Northwestern University, n = 5,812) and the Gene Environment Association Studies (GENEVA) in type 2 diabetes, which comprised participants from the Nurses’ Health Study (NHS, n = 3,314) and the Health Professional Follow-up Study (HPFS, n = 2,497). White participants in the NUgene study were genotyped on the Illumina 660W-Quadv1_A BeadChip platform, and African-American participants were genotyped on the Illumina 1M-Duo Beadchip platform [33]. All participants in the GENEVA study were genotyped on the Affymetrix 6.0 platform. The datasets for the analyses described in this manuscript were obtained through dbGaP (accession numbers phs000091.v2.p1 and phs000237.v1.p1). Details regarding the workflow and quality control decisions are described on the respective dbGaP webpages for each study.

Our objective was to identify SNPs associated with type 2 diabetes in two or more independent samples across platforms, as independent replication provides the strongest protection against false discovery. The four samples were first stratified by race/ethnicity to avoid confounding by population stratification, and 6 homogenous samples with a minimum sample size of 100 were identified for analysis (see Results and S1 Table). SNPs within the *MARCH1* region of chromosome 4 (from position 165,300,000 to 164,445,000 of the GRCh37.p13 gene assembly) were considered. The number of SNPs in common across the groups was enumerated, given differing platforms, differing QC decisions across studies, and exclusion of SNPs with extreme allele frequency, to account for the potential for replication across studies/platforms.

### Statistical analyses

All mouse data were analyzed in SAS Enterprise Guide 7.1 (SAS Institute, Cary, NC). Body weight and IPGTT were analyzed using a repeated-measures mixed-model ANOVA including genotype, time, and their interaction. Visceral adiposity as a proportion of body weight was analyzed separately for each sex using a one-way ANOVA with genotype as the main effect and age as a covariate. The results of the flow cytometric studies of the adipose SVF were analyzed by mixed ANOVA model including genotype, age, and genotype*age interactions. When appropriate, Tukey’s test was used to adjust for multiple comparisons.

The association between SNPs and type 2 diabetes was assessed using a log-additive logistic regression model, within each group. Figures were generated using the R package snp.plotter. Additional details are provided in the Results section. Overall, 497 SNPs were considered across the MARCH1 locus; only those SNPs shared between at least two platforms were considered.

**Fig 1:**
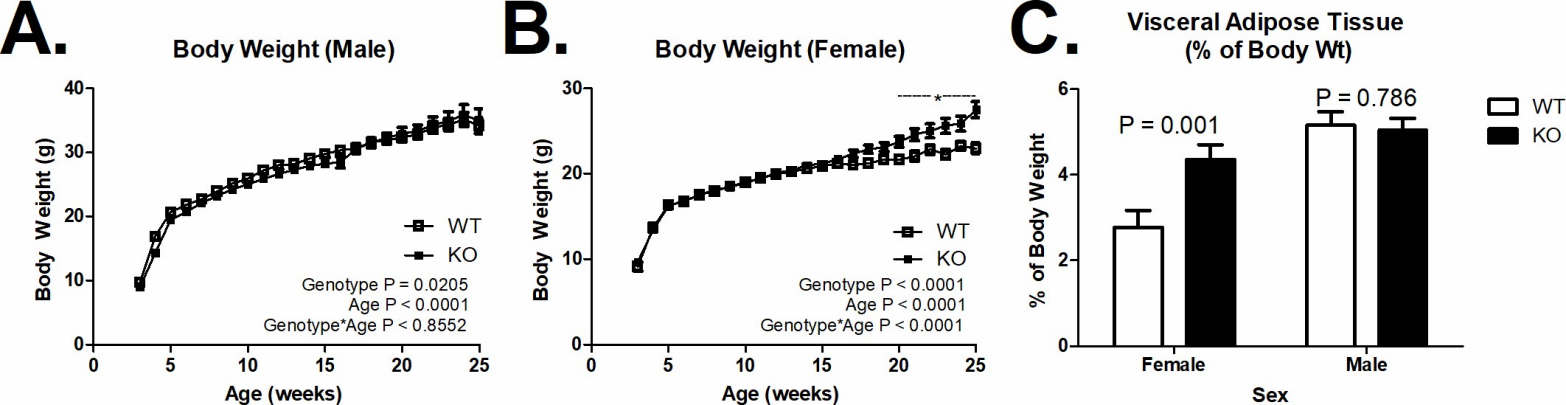
Body weight and visceral adiposity. (A) Increased body weight in female mice MARCH1-deficient mice (KO) compared to wildtype mice (WT) on a chow diet after 20 weeks of age (WT n = 10–33; KO n = 12–41). (B) MARCH1-deficiency did not affect the age-related increase in body weight in male mice (WT n = 8–19; KO n = 7–28). (C) Increased visceral adiposity in female, but not male mice KO mice. Number (*n*) is indicated within bars). *indicates significant difference P < 0.05. Data are plotted at the mean +/- SEM.

**Fig 2:**
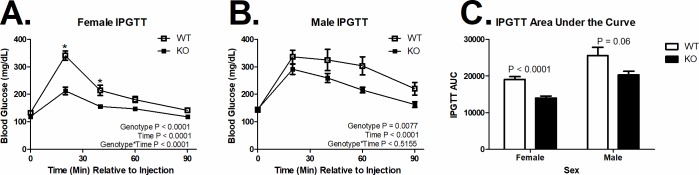
Glucose tolerance. (A) MARCH1 deletion (KO) decreased glucose clearance in female (WT n = 10, KO n = 12) and (B) male (WT n = 8, KO n = 7) mice. (C) Area under the glucose tolerance curve by sex and genotype (*n* is indicated within bars). *Indicates significant difference at that time point (P < 0.05). Data are plotted at the mean +/- SEM.

**Fig 3:**
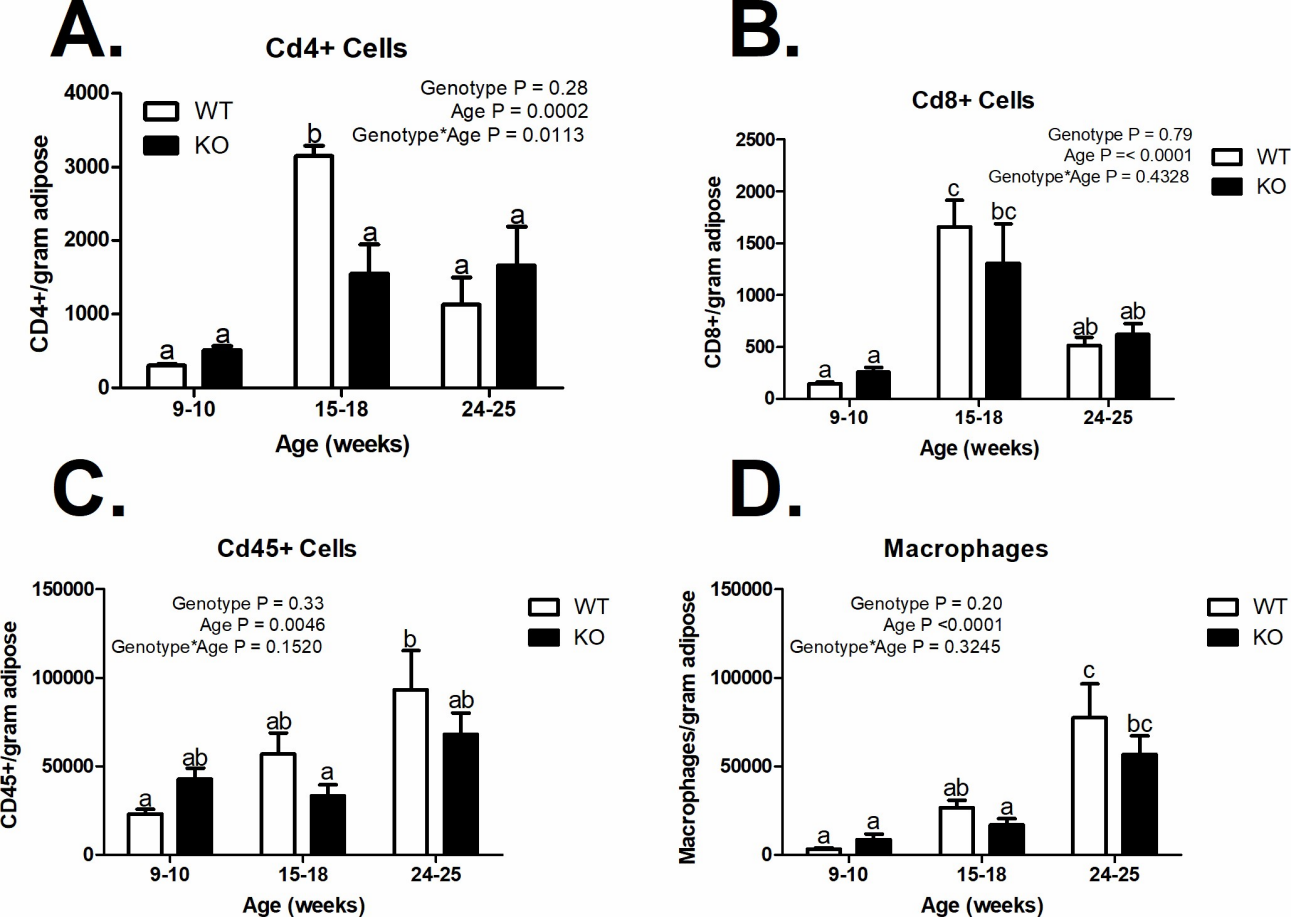
Immune cell infiltration of visceral adipose tissue. (A) CD4+ cells (*n* = 3), (B) CD8+ cells (*n* = 3), (C) CDd45+ cells (*n* = 3–6), and (D) Macrophages/gram of visceral adipose tissue (*n* = 3–6). *a*, *b*, *c* = bars that share a common superscript do not differ significantly (P > 0.05). The numbers are based on flow cytometric analyses using the gating and scheme shown in S1 Fig. Data are plotted at the mean +/- SEM.

**Fig 4:**
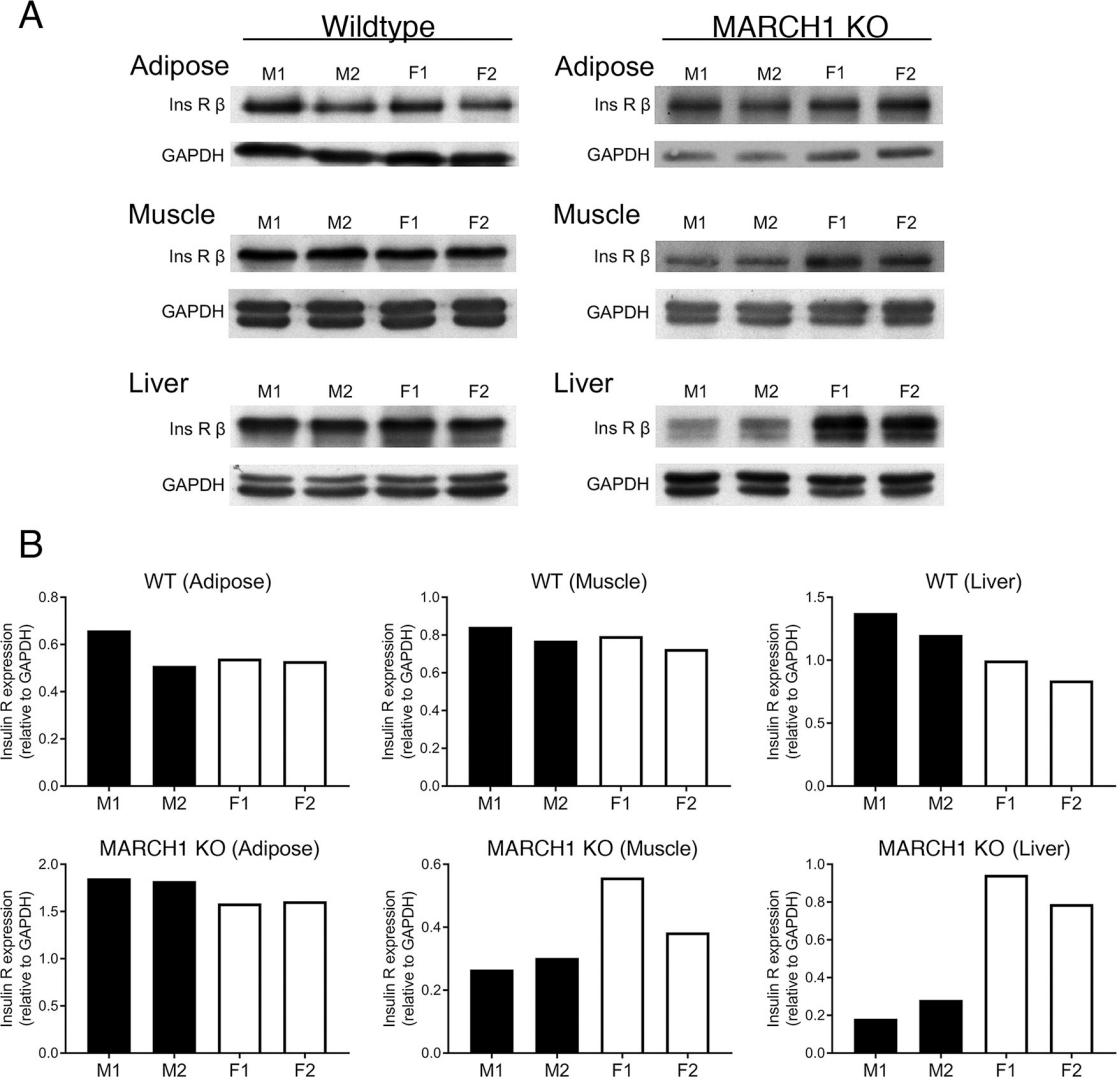
Comparison of insulin receptor levels in female *versus* male mice. Proteins from adipose tissue, skeletal muscle, and liver from either WT or MARCH1 KO mice were isolated, separated by SDS-PAGE, and immunoblotted using antibodies recognizing insulin receptor β-chain and GAPDH (as a loading control). Each group consisted of two male and two female littermates. (A) Immunoblots showing the expression levels of insulin receptor β-chain (Ins R β) and GAPDH present in each sample. (B) Immunoblots were scanned by laser densitometry and band intensity was quantified. The expression level of insulin receptor β-chain was plotted relative to the amount of GAPDH present in each sample.

The data used to produce the graphs in Figs 1–4 are housed in a public repository. The data are deposited within DRYAD (datadryad.org) with the accession number: doi:10.5061/dryad.r34k1.

## Results

### MARCH1 deficiency results in sex-specific changes in body weight and adiposity

Body weight was similar throughout time in male KO *versus* WT mice (Fig 1A; P > 0.05). However, MARCH1 deficiency increased body weight in female mice from 20–25 weeks of age compared to WT female mice (Fig 1B; P < 0.05). This increase in body weight may be associated with increased adiposity, as the ratio of visceral adipose tissue to body weight was higher in female MARCH1 KO mice than in MARCH1 WT mice (Fig 1C; P = 0.001). In line with the lack of body weight change, MARCH1 deficiency did not affect visceral adiposity in male mice (P = 0.79).

### MARCH1 deficiency increases glucose clearance

MARCH1 deficiency did not alter basal blood glucose (P > 0.5). However, MARCH1 deficiency improved glucose clearance assessed by IPGTT in both female (Fig 2A; P < 0.0001) and male (Fig 2B; P = 0.0077) mice. In fact, MARCH1 deficiency decreased the area under the curve (AUC) during the IPGTT by 26.7% (Fig 2C; P < 0.0001) in female mice and 20.5% (P = 0.06) in male mice.

### Leukocyte numbers in VAT are unaffected by MARCH1

The increased visceral adipose tissue accumulation in MARCH1 KO females prompted us to study adipose tissue inflammation. We performed flow cytometric analysis of the stromal-vascular fraction (SVF) purified from VAT of MARCH1 KO and WT female mice to determine the extent of leukocyte infiltration. S1 Fig depicts a typical gating scheme for analysis of hematopoietic lineage cells in SVF. Total SVF leukocytes, CD4+ cells, CD8+ cells, and macrophages did not differ between KO and WT mice, irrespective of age (Fig 3). As expected, the MARCH1 KO macrophages expressed higher levels of MHC-II (S1 Fig). Data are plotted at the mean +/- SEM.

### MARCH1 deficiency produces greater rescue of insulin receptor levels in females

To test whether the differences observed in female *versus* male mice could be explained, at least in part, by differential impact of MARCH1 deficiency on insulin receptor levels, immunoblots were performed with protein lysates from adipose, skeletal muscle, and liver (Fig 4). Receptor levels in tissues from female and male littermates were compared for wildtype or MARCH1 KO mice. In wildtype mice, the receptor levels were comparable in both sexes. In the absence of MARCH1, however, female mice had higher levels of the insulin receptor than males in the liver and to a lesser extent, in muscle; no difference was observed in adipose tissue.

### MARCH1 and type 2 diabetes in human populations

Our findings in mice, along with published genetic associations studies [28, 29], implicating MARCH1 in metabolic disease, prompted us to test for an association between *MARCH1* and type 2 diabetes in humans using GWAS datasets. Within the NUgene set, there were 139 non-Hispanic whites and 1,354 non-Hispanic blacks available for analysis. The NWU sample included 1,134 non-Hispanic whites and 242 non-Hispanic blacks. The HPFS included 2,397 non-Hispanic whites, and the NHS included 3,222 non-Hispanic whites (S1 Table). Other racial/ethnic groups were not sufficiently represented in these studies to allow for meaningful analyses. These six groups were then described in terms of case-control composition, sex, and family history of type 2 diabetes (S2 Table).

Logistic regression results are displayed graphically by sub-study (S2–S4 Figs). Complete results are also shown in tabular format, including minor allele frequency, odds-ratio for type 2 diabetes case status, raw *p*-value, Benjamini and Hochberg corrected *p*-value, and Bonferroni corrected *p*-value (S1 File). A total of 497 SNPs were available in at least two samples. Briefly, the number of SNPs with association (*p*<0.05, uncorrected for multiple comparisons) in each sample was as follows: HPFS, 15; NHS, 9; VU 660W platform, 5; NWU 660W platform, 6; VU 1M platform, 13; and NWU 1M platform, 26, though none were significant after correction for multiple comparisons (S1 File). Seven SNPs had significant associations (uncorrected) with type 2 diabetes in more than one sample, though not all of these associations were consistent across samples with respect to the direction of the association (Table 1). Two SNPs were found to be associated (*p*<0.05) with increased odds of type 2 diabetes in two analysis groups; rs12500778 in NHS and NWU, and rs17578337 in VU Whites and VU Blacks (Table 1). Both SNPs are intronic and lie at distance of ≈172 kb from each other.

**Table 1:** MARCH1 SNPs that are significantly associated with type 2 diabetes in more than one study.

		Study and Platform^a^																	
		HPFS_Affy			NHS_Affy			VU_660W			NWU_660W			VU_1M			NWU_1M		
SNP	Location^b^	MAF	OR	Raw *p*	MAF	OR	Raw *p*	MAF	OR	Raw *p*	MAF	OR	Raw *p*	MAF	OR	Raw *p*	MAF	OR	Raw *p*
rs13105536	164790122	28	0.98	0.8113	27.1	0.98	0.68329	31.3	0.93	0.78779	27.6	1.21	0.04539	6	0.64	0.00787	4.8	0.44	0.056
rs17044105	164887559	2.3	1.49	0.03967	2.1	0.7	0.04321	.	.	.	.	.	.	.	.	.	.	.	.
rs12500778	164939833	3.7	0.95	0.71279	4.2	1.32	0.02804	.	.	.	.	.	.	35.3	1.03	0.73125	35.1	1.98	0.0014
rs10517789	164946712	2.6	1.57	0.01338	2.6	0.62	0.00408	.	.	.	.	.	.	.	.	.	.	.	.
rs1494284	165016836	.	.	.	.	.		45.3	1.86	0.02901	46.1	1.17	0.06607	22.6	1.05	0.57169	23.1	0.64	0.0482
rs6821574	165021304	.	.	.	.	.	.	44.9	1.94	0.0193	46.5	1.16	0.07498	37.2	0.95	0.51311	38.4	0.66	0.0287
rs17578337	165111724	28.5	0.91	0.13834		30.1	1.05	0.39701		30.2	1.94	0.03907	29.6	1.16	0.09526		7.7	1.35	0.03963	8.7	0.76	0.3962

OR greater than 1 (p<0.05) in orange; OR less than 1 (p<0.05) in blue; Raw p<0.05 in green

HPFS, Health Professional Follow-up Study; NHS, Nurses’ Health Study; VU, Vanderbilt University; NWU, Northwestern University; SNP, Single-nucleotide polymorphism; MAF, minor allele frequence; OR, odds ratio

^a^Desciption of the studies and platforms is provided in the Materials and Methods

^b^SNP location on chromosome 4

Given the sex differences we observed in our mice with respect to VAT accumulation, we stratified our data based on sex and BMI for the top 7 hits. Using a log-additive logistic regression model, none of the SNPs were significantly associated with sex or BMI, nor did age modify the association in these studies (P > 0.05). Further, genotype did not modify the association between BMI and type 2 diabetes (P > 0.05).

## Discussion

Until recently, our knowledge of MARCH1 function came from the study of its role in antigen presentation to T cells [23, 34]. However, at least in overexpression studies, MARCH1 could regulate other molecules, as well [21]. Nagarajan, *et al*. recently found that MARCH1 causes degradation of the insulin receptor. MARCH1 loss improved insulin sensitivity in male and female mice on a normal chow diet, and MARCH1 did not alter weight gain or adiposity in male mice on chow diet [27]. We made similar findings in male mice, but noted that female chow-fed MARCH1 KO mice gained more weight and had more VAT than WT controls. The enhanced glucose uptake and resulting lipogenesis may explain the increased adiposity in female mice, but this same mechanism would be expected to induce similar effects in male mice. The increased adipose tissue lipid storage in females would reduce ectopic fat deposits and improve insulin action [35].

Sex differences in adipose tissue accumulation and function are multifactorial and many potential mechanisms may explain how MARCH1 exerts its sex-specific effect(s) [36, 37]. Both sexes of MARCH1 KO mice show improved glucose handling with loss of MARCH1, indicating that MARCH1 targets the insulin receptor in either case. However, it is possible that the magnitude of the MARCH1 effect on insulin receptor levels is more pronounced in females and could vary in different tissues. Indeed, when we compared insulin receptor levels between sexes in the absence of MARCH1, there were markedly higher levels of insulin receptor expression in the liver of female MARCH1 KO mice. This trend was less pronounced in skeletal muscle and not apparent in adipose tissue. Furthermore, it is known that visceral (perigonadal) adipocytes from female mice are more sensitive to insulin, in terms of activation of signaling cascades downstream for the insulin receptor and induction of lipogenesis [38]. Notably, this increased sensitivity in female adipocytes is not correlated with changes in insulin receptor expression levels [38]. Therefore, MARCH1-mediated downregulation of the insulin receptor may be greater in some tissues in female mice (and therefore MARCH1 loss leads to a greater restoration of insulin receptor levels), and/or an equivalent degree of insulin receptor upregulation with MARCH1 loss between sexes may have greater metabolic consequences in females; these possibilities remain to be fully tested, as does the manner in which sex differences impact the effect of MARCH1 on the insulin receptor. Regardless, the MARCH1 knockout mouse may be useful to identify sex-specific metabolic regulation or test for mechanisms regulating metabolic homeostasis in the absence of dietary stress.

In addition to the discovery that MARCH1 regulates the insulin receptor, it is possible that MARCH1 also impacts metabolism through its regulation of antigen presentation. Macrophages express relatively high levels of MARCH1 [22] and have come to occupy, along with adipocytes, a central position in immune homeostasis in adipose tissue [16, 39]. However, we did not detect obvious changes in leukocyte accumulation in adipose +/- MARCH1. Nonetheless, our findings warrant further analysis of the role for MARCH1 in metabolic substrate use and demands of different tissues.

The importance of MARCH1 in adipose tissue and liver was likely obscured by its relatively low expression in non-lymphoid tissues [21]. MARCH1 expression in APCs is regulated by multiple mechanisms; its levels generally decrease in pro-inflammatory settings and increase under anti-inflammatory conditions [24–26, 31, 40, 41]. Further, MARCH1 is an inherently unstable protein that is difficult to detect even in APCs [24, 31]. Though MARCH1 expression is relatively low in typical insulin-responsive tissues, this low level is clearly important to regulate the insulin receptor [27]. It is notable that MARCH1 mRNA in the liver is decreased by insulin treatment and that its levels increase in obese adipose tissue [27]. Thus, MARCH1 is broadly expressed, regulated by key metabolic hormones, and is a central regulator of insulin receptor homeostasis.

Two previous studies reported an association of *MARCH1* with metabolic phenotypes in humans (type 2 diabetes and BMI; [28, 29]). Our human candidate gene investigation was not strongly supportive after correction for multiple testing, though two SNPs were nominally associated (p<0.05) with increased odds of type 2 diabetes in independent samples/platforms (Table 1). Whether these findings are indicative of causal variants or false discovery is unknown, since the associations were not statistically significant according to our significance threshold adjusted for multiple testing. We have made our results available so that other research groups may investigate further. The fact that the genetic associations reported thus far have all been in Asian populations, which were not part of our study, may indicate that *MARCH1*’s impact on metabolism varies by race/ethnicity.

The discovery that MARCH1 regulates glucose handling provides a new avenue to explore metabolic regulation and disease. The fact that, in mice, MARCH1 has sex-specific effects on lipid storage further indicates that the study of MARCH1 will yield insights into the differences between males and females with respect to metabolic diseases. Lastly, it will be important to leverage the MARCH1 KO mouse system to explore MARCH1’s dual roles in metabolism and immune regulation, and determine potential links between these processes.

## Supporting information

S1 File Analysis of *MARCH1* SNPs for association with type 2 diabetes. Excel file containing the association data for all SNPs tested for association with type 2 diabetes. Abbreviations and legend are provided at the bottom of the table.
(XLSX)

S1 Fig Representative gating scheme for flow cytometric analysis of immune leukocytes in the stromal-vascular fraction (SVF) of visceral adipose tissue. **(**A) Light-scatter gating of the SVF stained with an isotype control (negative control) antibody. (B) SVF stained with anti-CD45 antibody. (C) CD11b and F4/80 staining of CD45+ cells. (D) MHC-II expression on the CD11b, F4/80 double-positive cells from wildtype (blue) and MARCH1-deficient mice. (E-G) Gating scheme for CD4+ and CD8+ T cells. (E) SVF stained with isotype control antibody. (F) CD45+ cell gate shown with a threshold applied so that the CD45-negative cells are not displayed. (G) CD4 and CD8 staining of the CD45+ cells.
(PDF)

S2 Fig Graphic representation of associations between each SNP and type 2 diabetes across the MARCH1 gene region, among white individuals on the 660W platform. SNP location is plotted on the x-axis, and the -ln(p-value) is on the y-axis. Up-pointing triangles indicate increased odds of type 2 diabetes, and down-pointing triangles indicate decreased odds of type 2 diabetes. Red triangles represent data from Northwestern University, and black triangles represent data from Vanderbilt University.
(PDF)

S3 Fig Graphic representation of associations between each SNP and type 2 diabetes across the MARCH1 gene region, among African American individuals on the 1M platform. SNP location is plotted on the x-axis, and the -ln(p-value) is on the y-axis. Up-pointing triangles indicate increased odds of type 2 diabetes, and down-pointing triangles indicate decreased odds of type 2 diabetes. Red triangles represent data from Northwestern University, and black triangles represent data from Vanderbilt University.
(PDF)

S4 Fig Graphic representation of associations between each SNP and type 2 diabetes across the MARCH1 gene region, among white individuals on the Affymetrix platform. SNP location is plotted on the x-axis, and the -ln(p-value) is on the y-axis. Up-pointing triangles indicate increased odds of type 2 diabetes, and down-pointing triangles indicate decreased odds of type 2 diabetes. Blue triangles represent data from the Health Professionals Follow-up Study, and red triangles represent data from the Nurse’s Health Study.
(PDF)

S1 Table Number of subjects in each racial/ethnic group according to study and genotyping platform. (PDF)

S2 Table Groups for preliminary analysis of MARCH1 SNPs and type 2 diabetes, grouped by race/ethnicity and genotyping platform. (PDF)
