# Supplementary figures and images for: The E3 ubiquitin ligase MARCH1 regulates glucose-tolerance and lipid storage in a sex-specific manner
Source: PLoS One. 2018 Oct 24;13(10):e0204898. doi: 10.1371/journal.pone.0204898 (PMC6200199; doi:10.1371/journal.pone.0204898)

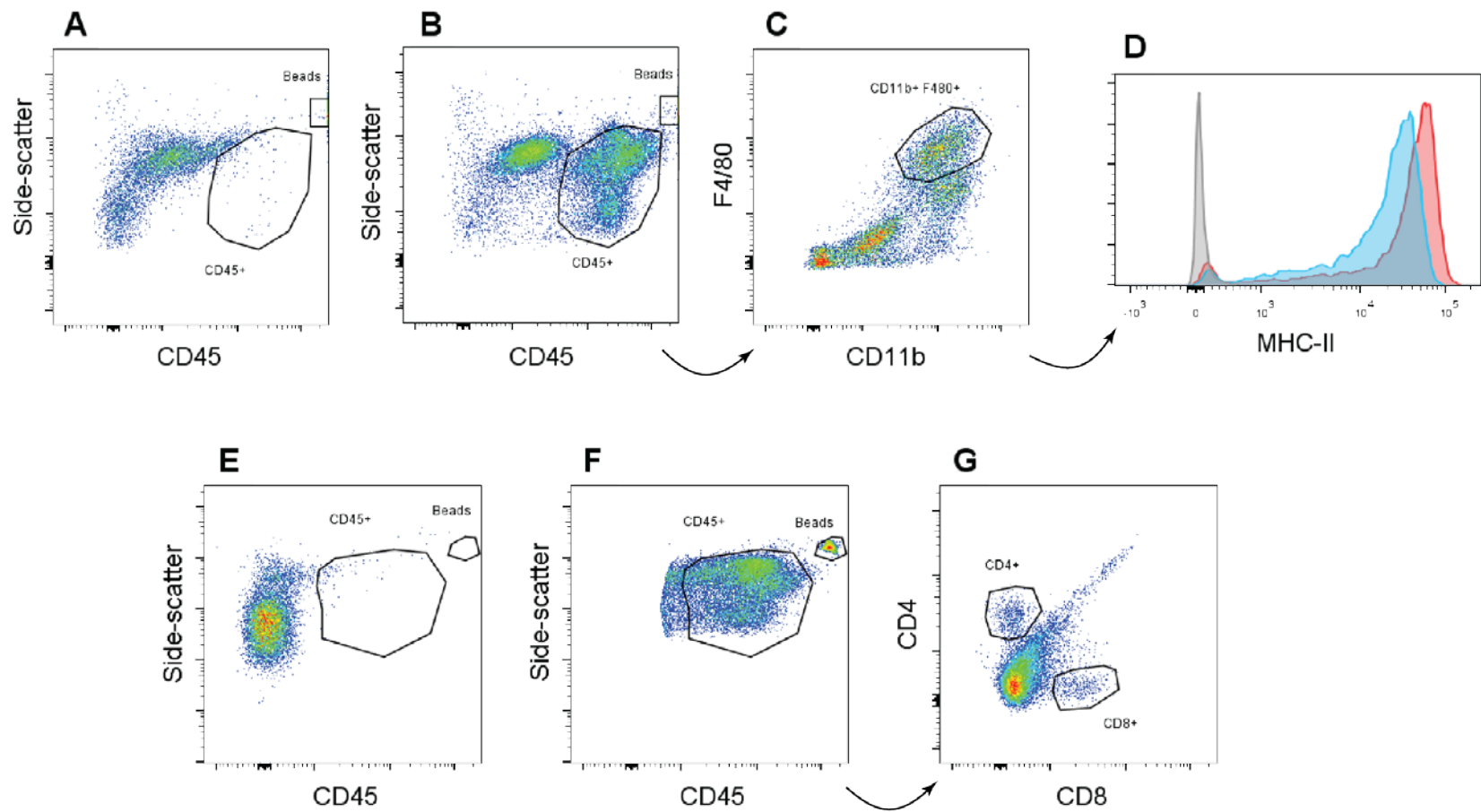

Supplement: S1 Fig — (A) Light-scatter gating of the SVF stained with an isotype control (negative control) antibody. (B) SVF stained with anti-CD45 antibody. (C) CD11b and F4/80 staining of CD45+ cells. (D) MHC-II expression on the CD11b, F4/80 double-positive cells from wildtype (blue) and MARCH1-deficient mice. (E-G) Gating scheme for CD4+ and CD8+ T cells. (E) SVF stained with isotype control antibody. (F) CD45+ cell gate shown with a threshold applied so that the CD45-negative cells are not displayed. (G) CD4 and CD8 staining of the CD45+ cells. (PDF) [file pone.0204898.s002.pdf]

# MARCH1, 660W platform, Whites

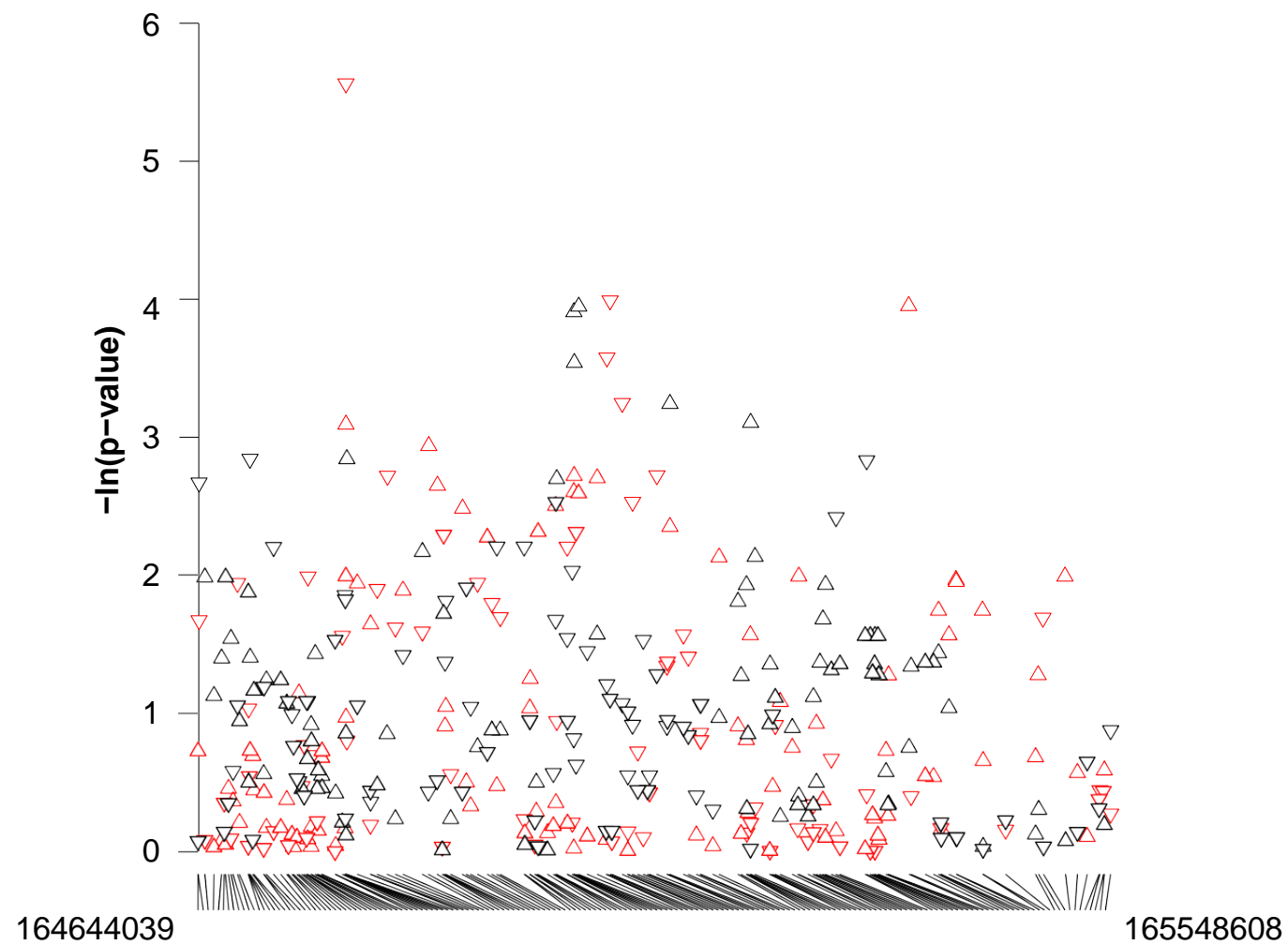

Physical Distance: 904.6 kb

▼ NWU

▼ VU

Supplement: S2 Fig — SNP location is plotted on the x-axis, and the -ln(p-value) is on the y-axis. Up-pointing triangles indicate increased odds of type 2 diabetes, and down-pointing triangles indicate decreased odds of type 2 diabetes. Red triangles represent data from Northwestern University, and black triangles represent data from Vanderbilt University. (PDF) [file pone.0204898.s003.pdf]

# MARCH1, 1M platform, African Americans

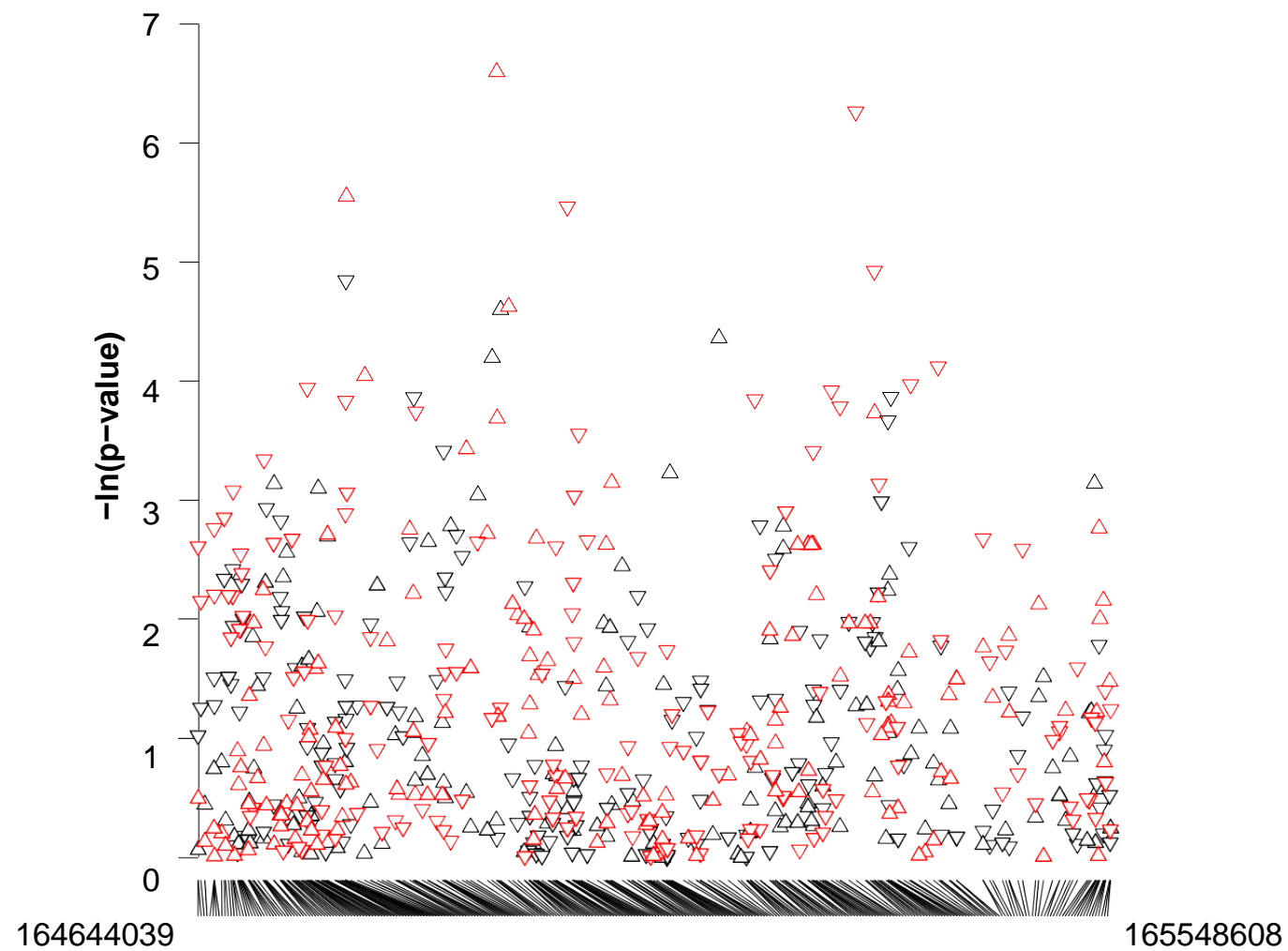

Physical Distance: 904.6 kb

▽ VU

▽ NWU

Supplement: S3 Fig — SNP location is plotted on the x-axis, and the -ln(p-value) is on the y-axis. Up-pointing triangles indicate increased odds of type 2 diabetes, and down-pointing triangles indicate decreased odds of type 2 diabetes. Red triangles represent data from Northwestern University, and black triangles represent data from Vanderbilt University. (PDF) [file pone.0204898.s004.pdf]

# MARCH1, Affy platform, Whites

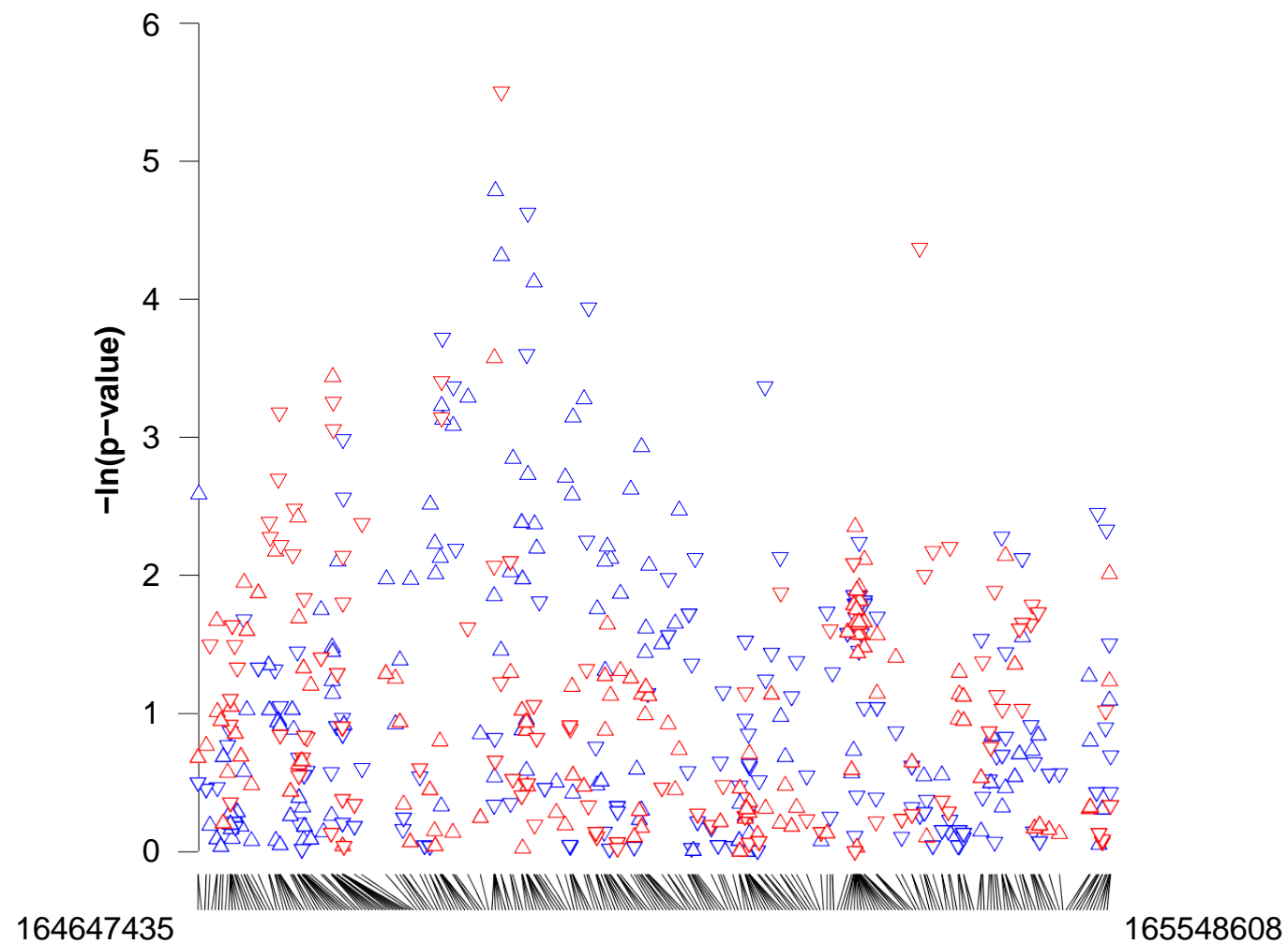

Physical Distance: 901.2 kb

▼ HPFS      ▼ NHS

Supplement: S4 Fig — SNP location is plotted on the x-axis, and the -ln(p-value) is on the y-axis. Up-pointing triangles indicate increased odds of type 2 diabetes, and down-pointing triangles indicate decreased odds of type 2 diabetes. Blue triangles represent data from the Health Professionals Follow-up Study, and red triangles represent data from the Nurse’s Health Study. (PDF) [file pone.0204898.s005.pdf]
