# Supplementary material for: The E3 ubiquitin ligase MARCH1 regulates glucose-tolerance and lipid storage in a sex-specific manner
Source: PLoS One. 2018 Oct 24;13(10):e0204898. doi: 10.1371/journal.pone.0204898 (PMC6200199; doi:10.1371/journal.pone.0204898)
Supplement: S1 Table — (PDF) [file pone.0204898.s006.pdf]

S1 Table. Number of subjects in each racial/ethnic group according to study and genotyping platform.

	Total (n=9169)					
	NUgene[a] (n=3357)				GENEVA (n=5812)	
	Vanderbilt (n=1951)		Northwestern (n=1406)		HPFS (n=2497)	NHS (n=3315)
**Race/ethnicity**	660W[b] (n=597)	1M (n=1354)	660W (n=1159)	1M (n=247)	Affy (n=2497)	Affy (n=3315)
White, not Hispanic	**139**	0	**1134**	0	**2397**	**3222**
White, Hispanic	2	0	25	0	0	34
Black, not Hispanic	0	**1354**	0	**242**	25	26
Black, Hispanic	0	0	0	5	0	2
American Indian	0	0	0	0	0	14
Asian	0	0	0	0	25	17
Other	0	0	0	0	50	0
Unknown/missing	456	0	0	0	0	0

Underlined numbers indicate subjects included in the current analysis

HPFS, Health Professional Follow-up Study; NHS, Nurses' Health Study

[a]Indicates study

[b]Indicates genotyping platform
