# Supplementary material for: The E3 ubiquitin ligase MARCH1 regulates glucose-tolerance and lipid storage in a sex-specific manner
Source: PLoS One. 2018 Oct 24;13(10):e0204898. doi: 10.1371/journal.pone.0204898 (PMC6200199; doi:10.1371/journal.pone.0204898)
Supplement: S2 Table — (PDF) [file pone.0204898.s007.pdf]

**S2 Table. Groups for preliminary analysis of MARCH1 SNPs and type 2 diabetes, grouped by race/ethnicity and genotyping platform**

| | White, not Hispanic (n=6892) | | | | | | | | Black, not Hispanic (n=1596) | | | |
|---|---|---|---|---|---|---|---|---|---|---|---|---|
| | Vanderbilt[a]/660W[b] (n=139) | | Northwestern/660W (n=1134) | | HPFS/Affy (n=2397) | | NHS/Affy (n=3222) | | Vanderbilt/1M (n=1354) | | Northwestern/1M (n=242) | |
| Cases (%) | **Cases** | **Controls** | **Cases** | **Controls** | **Cases** | **Controls** | **Cases** | **Controls** | **Cases** | **Controls** | **Cases** | **Controls** |
| | **99 (71.2)** | **40** | **530 (46.7)** | **604** | **1116 (46.6)** | **1281** | **1466 (45.5)** | **1756** | **598 (44.2)** | **756** | **153 (63.2)** | **89** |
| Females (%) | 45 (45.5) | 22 (55.0) | 233 (44.0) | 337 (55.8) | 0 (0.0) | 0 (0.0) | 1466 (100.0) | 1756 (100.0) | 373 (62.4) | 501 (66.3) | 97 (63.4) | 68 (76.4) |
| Family history of diabetes No (%) | 47 (47.5) | 28 (70.0) | 0 | 0 | 635 (56.9) | 1009 (78.8) | 741 (50.5) | 1370 (78.0) | 128 (21.4) | 479 (63.4) | 0 | 0 |
| Yes (%) | 48 (48.5) | 5 (12.5) | 460 (86.8) | 0 | 481 (43.1) | 272 (21.2) | 725 (49.5) | 386 (22.0) | 456 (76.3) | 204 (27.0) | 143 (93.5) | 0 |
| Unknown/missing (%) | 4 (4.0) | 7 (17.5) | 70 (13.2) | 604 (100.0) | 0 | 0 | 0 | 0 | 14 (2.3) | 73 (9.7) | 10 (6.5) | 89 (100.0) |

HPFS, Health Professional Follow-up Study; NHS, Nurses' Health Study

[a]Indicates study

[b]Indicates genotyping platform
